# Games between stakeholders and the payment for ecological services: evidence from the Wuxijiang River reservoir area in China

**DOI:** 10.7717/peerj.4475

**Published:** 2018-03-08

**Authors:** Lin Shu

**Affiliations:** 1 Jiangsu Key Laboratory for Biodiversity and Biotechnology, College of Life Sciences, Nanjing Normal University, Nanjing, Jiangsu, China; 2 College of Environment, Quzhou University, Quzhou, Zhejiang, China

**Keywords:** Ecological services, Wuxijiang River reservoir area, Gambling, Ecological compensation

## Abstract

A gambling or “game” phenomenon can be observed in the complex relationship between sources and receptors of ecological compensation among multiple stakeholders. This paper investigates the problem of gambling to determine payment amounts, and details a method to estimate the ecological compensation amount related to water resources in the Wuxijiang River reservoir area in China. Public statistics and first-hand data obtained from a field investigation were used as data sources. Estimation of the source and receptor amount of ecological compensation relevant to the water resource being investigated was achieved using the contingent valuation method (CVM). The ecological compensation object and its benefit and gambling for the Wuxijiang River water source area are also analyzed in this paper. According to the results of a CVM survey, the ecological compensation standard for the Wuxijiang River was determined by the CVM, and the amount of compensation was estimated. Fifteen blocks downstream of the Wuxijiang River and 12 blocks in the water source area were used as samples to administer a survey that estimated the willingness to pay (WTP) and the willingness to accept (WTA) the ecological compensation of Wuxijiang River for both nonparametric and parametric estimation. Finally, the theoretical value of the ecological compensation amount was estimated. Without taking other factors into account, the WTP of residents in the Wuxi River water source was 297.48 yuan per year, while the WTAs were 3864.48 yuan per year. The theoretical standard of ecological compensation is 2294.39–2993.81 yuan per year. Under the parameter estimation of other factors, the WTP of residents in the Wuxi River water source area was 528.72 yuan per year, while the WTA was 1514.04 yuan per year. The theoretical standard of ecological compensation is 4076.25–5434.99 yuan per year. The main factors influencing the WTP ecological compensation in the Wuxi River basin are annual income and age. The main factors affecting WTA are gender and attention to the environment, age, marital status, local birth, and location in the main village.

## Introduction

Environmental services such as natural purification of water, erosion control, and habitat for wildlife are public goods that have value to society but are difficult to assign a market value to. In the relevant market, benefits provided by natural resources can be expressed as values to human well-being (Arrow et al., 1995; Costanza et al., 1997; Wackernagel et al., 1999; Ouyang, Wang & Miao, 1999; Daily et al., 2000; de Groot, Wilson & Boumans, 2002; Xu, Liu & Chang, 2013). Ecological compensation is the institutional arrangement for regulating and protecting the interests of stakeholders based on the protection and sustainable utilization of environmental services (Jin, Li & Zuo, 2007). Global drinking water resources face enormous challenges (Turner et al., 2003; World Health Organization, 2005), and ecological compensation is a vital mechanism by which water resources and land are equitably protected (Wunder, 2005; Shen & Gao, 2009; Lai, Wu & Yin, 2015). Compared with most countries in the world, the disparate systems and mechanisms in China make the relationship between the stakeholders of the ecological compensation of water resources more complex, with intertwined relationships. Source and receptor gambling is defined as a conflict of interest with compromise between sources and receptors, which is the key problem to be solved (Wang, Su & Cui, 2011; Zhang, Ming & Niu, 2017). Ecological compensation is divided into government compensation and market compensation. The majority of government compensation is relatively simple, while the market compensation is relatively diverse (Pretty & Ward, 2001; Zbinden & Lee, 2004; Ferraro, 2008). Due to the complexity regarding providing ecological compensation of water sources, one cannot simply implement ecological compensation according to the general principle of “whoever pollutes will pay.” Based on the theory of externality (Shen & He, 2002), compensation should start from an analysis of the beneficiaries of the watershed to identify who will compensate whom (Shen & Yang, 2004; Shen & Gao, 2009).

We can judge the subject and object of compensation through the division of powers. If the beneficiary object is determined, the beneficiary will be required to make compensation. If the social benefits or the beneficiary object cannot be determined, the government will make compensation (Qiao, Yang & Yang, 2012). Stakeholder analysis rules in ecological compensation state that based on the importance of initiative, decisiveness, and interest in each decision, the government, farmers, and enterprises can be defined as core stakeholders (Brown et al., 2014; Chen, 2014). Many scholars think that the government and residents in the upstream area of the water source are the compensable subjects (He, 2012). From the perspective of fairness and stability, residents should be compensated prior to the government receiving compensation. In terms of the goal of maximizing social wealth, the government is the ecological compensation object for the water source, not the residents. However, some scholars hold the opposite view that government is neither a beneficiary of ecological compensation nor a loser, so they should not be included in the scope (Wang et al., 2010).

In the region of study, the Wuxijiang River is the main water resource, which aids in regional sustainable development. In recent years, the rapid development of China’s economy has led to more and more serious environmental problems in Wuxijiang River, as the basin has the dual attributes of being both a water source and an economic development zone (Landell-Mills & Porras, 2002; Pagiola, Arcenas & Platais, 2004; Shen & Yang, 2004; Gao & Wen, 2004; Shen & Lu, 2004; Thapa, 2016) The issues of upstream environmental impacts and downstream effects may negatively affect environmental protections. To a large extent, these impacts are caused by the contradictory goals of economic development and ecological optimization. An issue that requires urgent attention is determining how to create ecological benefits both upstream and downstream of a water source while promoting stable development. At present, researchers mainly analyze how to make use of ecological compensation mechanisms to realize the comprehensive management of the ecological environment of the water source. One of the most important problems concerning the mechanism of ecological compensation is the standard of compensation acquisition, that is, the conflict between willingness to pay (WTP) and the willingness to accept (WTA) (Yang, Wang & Sun, 2014; Zhao, Li & Peng, 2016). There are many methods that attempt to account for the amount of ecological compensation that is appropriate for a given water source area (Ouyang, Wang & Miao, 1999; Research Group on China’s Ecological Compensation Mechanism and Policy, 2007; Ruan, Xu & Zhang, 2008; Fen, Wang & Yang, 2009; Lu & Ding, 2009; Sagona et al., 2016; Zhou, Sun & Cui, 2017). Through the investigation of WTP and WTA, lack of attention to the direct contributors and protectors of the ecosystem, or the inadequacy of compensation can be avoided, which are in turned caused by source and receptor gambling (Wunder, 2005).

The Wuxijiang River is the first water source to have protections legislated by the Zhejiang Provincial People’s Congress (Xie, 2003). Ecological protection and compensation involve many stakeholders. There is an administrative/subordinate relationship among the stakeholders, which can be regarded as a multi-level principal agent relationship. Under this multi-level principal agent relationship, the conflicts of interests between the main stakeholders—such as the government, industry, and the residents—are constantly escalating. The central and local governments have a distribution conflict of income rights for the development of resources, and hold different priorities concerning the promotion of development of the local economy. It is valuable to analyze the interests of all core stakeholders concerning regional water conservation. Using the Wuxijiang River in China as an example, this study investigates the residents’ WTP and the WTA as analyzed using the contingent valuation method (CVM) (Zhang et al., 2002; Zhang & Zhao, 2007). This method allowed an estimation of the amount of ecological compensation from water resources, as well as insight into how to better establish and improve equitable compensation mechanisms in the future.

## Research Region, Data Sources, and Methods

### Research region

**Figure 1:**
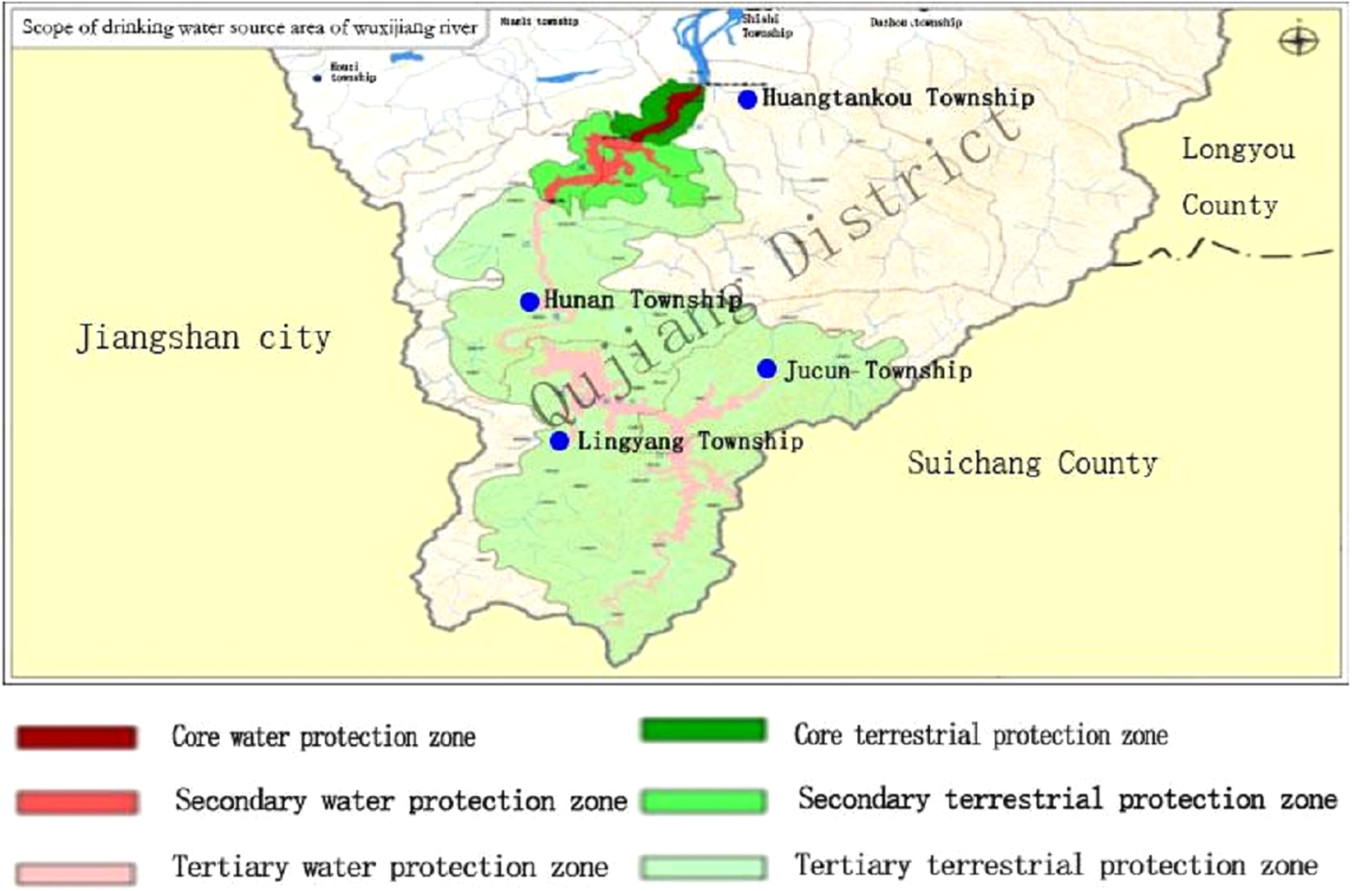
Administrative region division of the Wuxijiang River. The county, city, and district referred to herein in this article are all county-level units the Wuxijiang River reservoir was chosen as the research region. Its administrative region mainly involves the four townships of Hunan, Jucun, Lingyang, and Huangtankou, in the Qujiang district of Quzhou.

**Figure 2:**
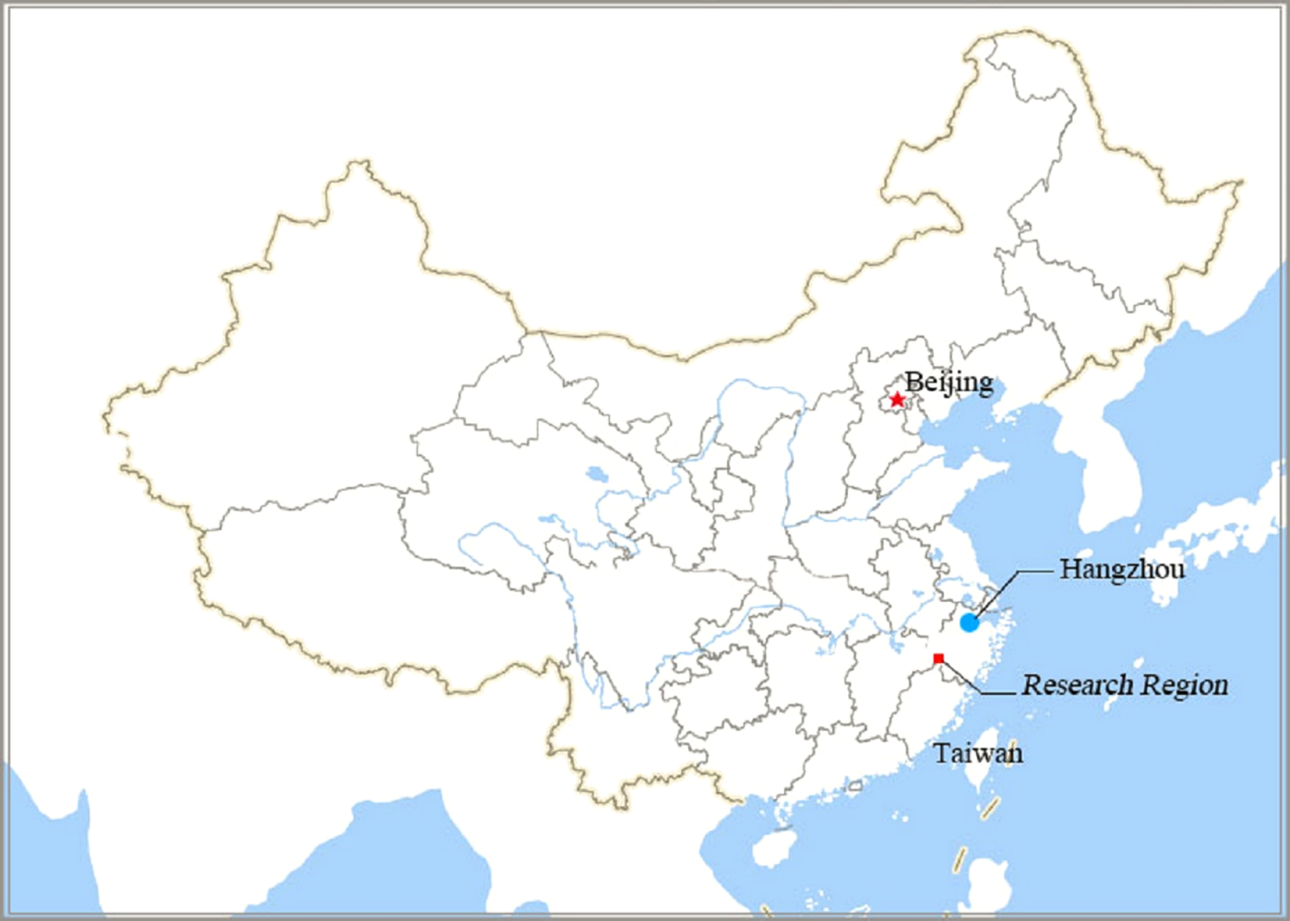
Location of research region on Map of China. Hangzhou is the capital city of Zhejiang province.

**Figure 3:**
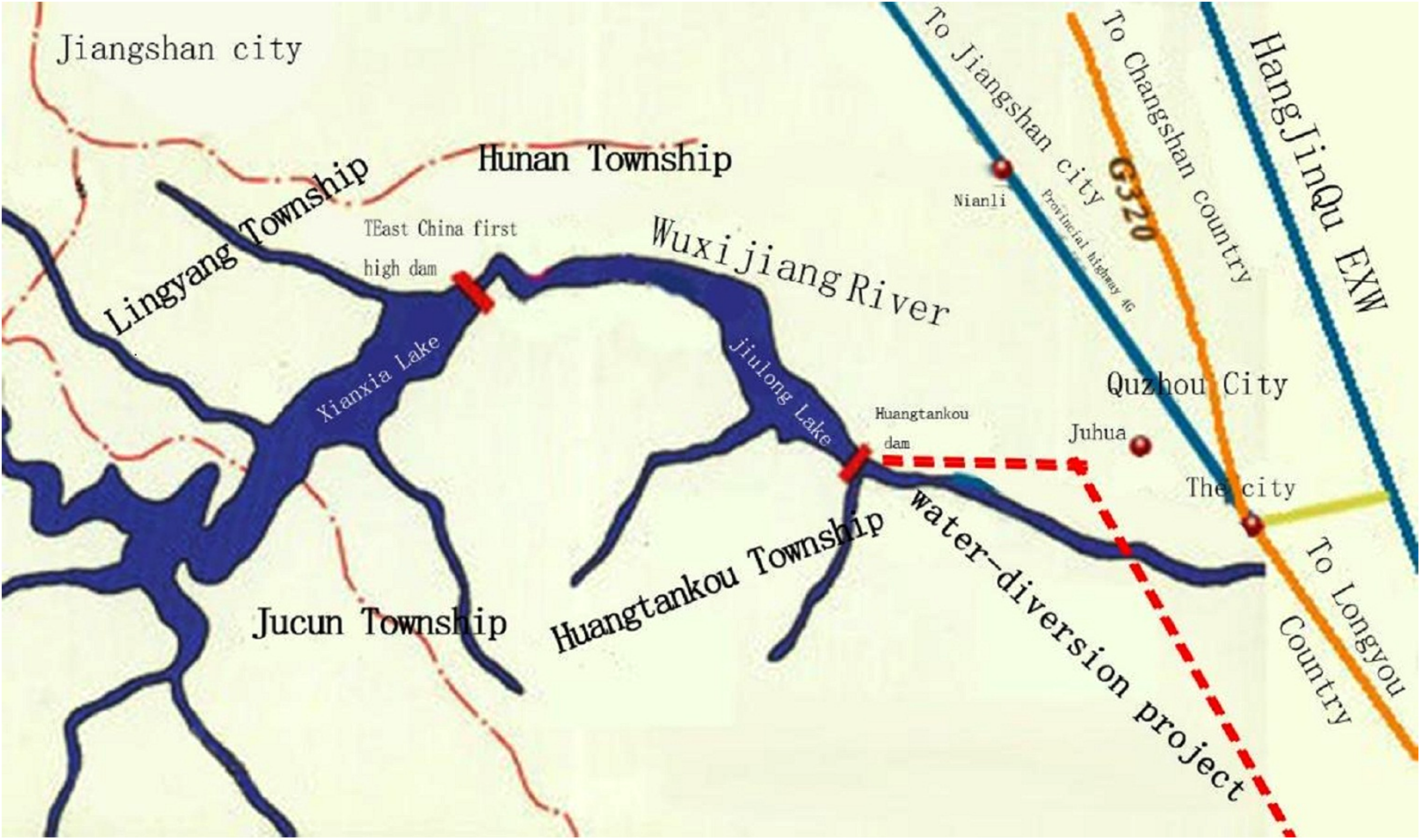
Watershed system of Wuxijiang River.

The Wuxijiang River reservoir was chosen as the research region. Its administrative region mainly involves the four townships of Hunan, Jucun, Lingyang, and Huangtankou, in the Qujiang district of Quzhou, as shown in Fig. 1. Its position in China is shown in Fig. 2. The watershed system of Wuxijiang River is shown in Fig. 3.

The Wuxijiang River watershed has reservoirs in Hunan and Huangtankou Townships, a primary tributary in Qujiang district, and a water diversion project in Wuxijiang River. The Wuxijiang River watershed has high ecological value and is a good source of quality fresh water. It not only has high forest coverage and abundant biological diversity, but also has a state-level wetland park. However, the Wuxijiang River watershed is facing ecological and environmental problems that are associated with a relatively fragile ecosystem, impacts and pollution from livestock and poultry breeding, negative environmental impacts from tourism and aquaculture, industrial pollution, and farm run-off.

### Data sources

Data were derived from public statistics and data collection through a household survey of 12 villages in four townships and 15 blocks on the Wuxijiang River in December 2015 by the project group. At least 30 households were randomly selected from each village for survey. The project group issued 385 surveys, of which 383 were returned and three were invalid. In total, there were 380 valid surveys. The contents of the survey included information about the economic conditions of farmers and householders, suggestions by the local government for ways to improve the ecological protection of the water source, and historical data. The urban community survey on respondents’ WTP for water received a total of 552 valid surveys. The contents included respondents’ personal information, family water use, their understanding of ecological protection of water sources, WTP for water, and the mode of payment.

### Research method

There are many methods to estimate the amount of compensation that should be granted for impacted ecological resources such as water (Liu, Xu & Zhang, 2006; Wunder, Engel & Pagiola, 2008; Wang, Wang & Liu, 2007; Li & Chen, 2003; Liu, Liu & Xu, 2014; Wei & Shen, 2011; Xie, Xi & Huang, 2014; Xiao et al., 2015). In order to effectively focus on the direct protection of water and contributors, the CVM (Ciriacy-Wantrup, 1947; Xu et al., 2003; Zhang & Zhao, 2007; Qiao, Cheng & Liu, 2016) was chosen for this research as a reference basis for the ecological compensation standard.

## Stakeholder Ecological Compensation and Their Benefits Via Gambling About the Wuxijiang River Water Source

### Analysis of stakeholders of ecological compensation in the water source area of the Wuxijiang River

Generally, the impacted research subjects analyzed are the government, industry, and the residents (Lami, Masetti & Neri, 2016). As the first government protected water source in Zhejiang province, Zhejiang provincial government and local government have implemented nearly 20 years of ecological protection policy. The Wuxijiang River power plant is the only remaining large industry. Most other industries have been closed or moved. To quantify the ecological compensation of the water source, we solve for the amount of gambling of the enterprises, government, and residents are willing to engage in. The Wuxijiang River power plant pays the water resources fees and the reservoir funds. There are no direct relationships between industry, local governments, and water sources. Therefore, the relationship between industry, government and residents is relatively simple. The only conflict of interest between the government and industry which needs to be coordinated is that of water use and supply. Therefore, the ecological compensation entities for Wuxijiang River are mainly the local governments and the residents.

### The primary impacts on residents in the water source area

There are three levels of water source protection; the core area, the secondary core area, and the water’s edge. Because water resources development occupies a large amount of land, regional environmental protection can lead to limitations in further developing industry, thus reducing the livelihood opportunities for local residents, which negatively affects economic development. The interests of residents in water areas need to be further determined, and require government departments to create effective coordination programs.

### The primary impacts on governments at all levels in Quzhou city, Qujiang district and township governments

There is a question as to which level of government is most impacted by claims of compensation throughout the watershed. This study argues that ecological compensation is mainly to internalize the relevant production costs so as to optimize ecosystem service function. For the ecological compensation of Wuxijiang River, the external costs are the investment cost of ecological protection and construction. Therefore, the government level most relevant to the target of ecological compensation is related to the compensation degree of the opportunity cost of development. Wuxijiang River is a tributary on the upper reaches of the source of the Qiantangjiang River, and development costs can be compensated in Quzhou city. With Quzhou city’s focus on ecological protection and construction, its GDP value will not have a great impact on Zhejiang Province. The loss of opportunity for the development of the Wuxijiang River’s water source protection area is fully shared across the area under the jurisdiction of the Quzhou city government (Yang, Cai & Zhang, 2017).

### Payment for gambling on environmental services in the Wuxijiang River source area

The main stakeholders’ benefit for playing the game with Wuxijiang River water resources includes the benefit games between the local government and the central government, local governments at all levels, and residents and the government.

The benefit game between the local government and the central government is reflected in the mismatch of the financial and administrative power controlling the water resource, which shows the trend of financial right above power. The status of central versus local governments differs greatly. The administrative power of the local government is passively increased. The interest’s game between the local governments at all levels is mainly reflected in that the financial and administrative rights do not match between the superiority and inferiority within the administrative hierarchy. If the county government manages directly, while giving the corresponding financial power, the system will be smoother (Song & Liu, 2005). The interest game between the residents and the government in the water source area is mainly caused by the misunderstanding of the compensation methods by residents in water sources. At present, the water source ecological compensation in China is basically government compensation. The mode of compensation is divided into two types: transfusion and hematopoiesis. The former means to give the compensation materials to the residents to sustain their basic lives, and the latter means the compensation materials given can further help them increase income and improve their living standards. The residents tend to prefer the transfusion type, hoping to direct compensation with money. They have low recognition of the hematopoietic compensation type such as policy compensation and industrial compensation (Gen et al., 2010), which leads to the lack of resources, insufficient compensation, and psychological satisfaction, resulting in an objective benefit game.

### Estimating compensation amount using CVM-based ecological methods

According to the results of the CVM survey, the ecological compensation standard of Wuxijiang River was determined using conditional value evaluation. The sample area included 15 blocks in the lower reaches of the Wuxijiang River Reservoir Area and the 12 villages in four towns in Wuxijiang River. In the two cases of nonparametric and parametric estimation, we estimated the WTP and WTA for ecological compensation in Wuxijiang River in order to obtain the ecological compensation theory value.

### Design of CVM survey

In the design of the survey, WTP and WTA were investigated. The survey on WTP covered 15 blocks in the Wuxijiang River water source. The detailed sample distribution is described in Table 1.

**Table 1:** WTP samples distribution.

Layer	Block	Sampling proportion (%)	Sample size
1st layer	Baidu village	5.25	29
2nd layer	Qiaotouwang village	5.43	30
3rd layer	Xiajiang village	5.43	30
4th layer	Shangjiang village	5.25	29
5th layer	Wang village	7.61	42
6th layer	Zaojiao village	5.07	28
7th layer	Yejia village	5.80	32
8th layer	Meijia village	6.88	38
9th layer	Zijing residential area (community)	8.15	45
10th layer	Jingui residential area (community)	7.25	40
11th layer	Wenjingyuan (community)	7.43	41
12th layer	Yulongwan area (community)	9.60	53
13th layer	Chongwenguolv (community)	7.61	42
14th layer	Juhuabin first residential area (community)	7.07	39
15th layer	Juhuabin second residential area (community)	6.16	34
Total	15 blocks	100.00	552

**Note:**

The survey on WTP covered 15 blocks in the Wuxijiang River water source.

The survey covered three areas. The first area included detailed respondent information, to describe and understand the basic social characteristics of the interviewees and provide the basis for the further analysis of the data. The detailed socio-economic characteristics of the WTP sample is described in Table 2.

**Table 2:** Socio-economic characteristics of sample of willingness to pay.

Features	Items	Sample size	Ratio (%)
Gender	Male	281	50.91
	Female	271	40.09
Age (years old)	Under 20	39	7.07
	20–30	74	13.41
	30–40	102	18.48
	40–50	117	21.2
	50–60	97	17.57
	Over 60	123	22.28
Family annual income (thousand yuan)	Less than 20	118	21.38
	20–30	94	17.03
	30–40	63	11.41
	40–50	45	8.15
	50–60	74	13.41
	60–70	29	5.25
	70–80	35	6.34
	Above 80	94	17.03
Occupation	State organizations, persons in charge in enterprises and institutions	59	10.69
	Professional and technical personnel	42	7.61
	Clerks and related personnel	17	3.08
	Commercial staff	22	3.99
	Service industry staff	44	7.97
	Agriculture, forestry, animal husbandry, and fishery workers	51	9.24
	Civil servants, teachers, journalists	35	6.34
	Medical staff, lawyers, financial practitioners	24	4.35
	Soldiers	2	0.36
	Other	256	46.38
Educational level	Primary school and below	150	27.17
	Junior middle school	108	19.57
	High school (including secondary school, vocational school)	112	20.29
	Junior college	62	11.23
	Undergraduate	110	19.93
	Graduate students (Master’s) and above	10	1.81

The second area was analyzed to better understand the use of water resources, including the price of water in Quzhou, the monthly water consumption of interviewees, and the level of concern about water-related environmental problem of the interviewees. This data was analyzed to understand if the local water quantity and water quality meet the overall demand, as well as to improve the overall understanding of environmental protection in the Wuxijiang River watershed.

A survey of WTA was conducted to investigate the 12 administrative villages with the shortest distance between Wuxijiang River reservoir in the four townships of Lingyang and Jucun townships in the upper reservoir, while Hunan Township and Huangtankou Township in the lower reservoir. The detailed socio-economic characteristics of WTA interviewees is described in Table 3.

**Table 3:** Socio-economic characteristics of sample of willingness to accept.

Features	Option	Sample size	Ratio (%)
Gender of interviewee	Male	206	54.21
	Female	357	45.79
Gender of householder	Male	352	92.63
	Female	28	7.37
Age of interviewee (years old)	Children (0–6)	0	0.00
	Juveniles (7–17)	3	0.79
	Youth (18–40)	30	7.89
	Middle age (41–65)	247	65.00
	Seniors (more than 66)	100	26.32
Education level of householder	Primary school and below	194	51.05
	Middle school	134	35.26
	High school	43	11.32
	University	9	2.37
Age of the householder (years old)	Children (0–6)	0	0.00
	Juveniles (7–17)	0	0.00
	Youth (18–40)	9	2.37
	Middle age (41–65)	257	67.63
	Seniors (more than 66)	114	0.30
Work situation	Fixed job	243	63.95
	Unfixed job	123	36.05
Have modern toilet	Yes	204	53.68
	No	176	46.32
Head of householder a local born	Yes	349	91.84
	No	31	8.16
Moved within five years	Yes	18	4.74
	No	362	95.26
Dwell near the main road	Yes	347	91.32
	No	33	8.68
Marital status	Unmarried	11	2.90
	Married	336	88.42
	Widowed	33	8.68
Home’s exterior	Mud room	75	19.74
	Single layer brick tile room	137	36.05
	Buildings	168	44.21
Is it cash from non-farm industry	Yes	24	6.32
	No	356	93.68

**Note**:

The detailed socio-economic characteristics of WTA interviewees.

## Empirical Research and Results

### Nonparametric estimation of the average WTP

Table 4 shows WTP for water source ecological compensation of the Wuxijiang River. The survey results of WTP were analyzed, and the expectation of the average WTP was computed on the basis of the value and frequency of WTP. The mathematical expectation model of discrete-time variable WTP was used in the computation of WTP (Kong, Xiong & Zhang, 2014; Guan et al., 2016), expressed as:

$${E_{{\rm{WTP}}}} = \mathop \sum \limits_i^m {B_i} \cdot {P_i} = 46.06\;{\rm{yuan}}/\left( {{\rm{year}} \cdot {\rm{household}}} \right)$$ (1)

where *B_i_* is the amount of tender, *P_i_* is the probability of the amount chosen by the interviewee, and *m* is the number of tenders that can be selected, which is set to 11 in this paper.

**Table 4:** WTP for water source ecological compensation of Wuxijiang River.

WTP (Yuan/household·month)	WTP (Yuan/household·year)	Frequency (household)	Positive WTP rate (%)	Total positive WTP rate (%)	WTP rate (%)	Total WTP rate (%)
0	0	255	–	–	46.2	46.2
10	120	111	37.37	37.37	20.11	66.3
20	240	54	18.18	55.56	9.78	76.09
30	360	23	7.74	63.3	4.17	80.25
50	600	56	18.86	82.15	10.14	90.4
80	960	4	1.35	83.5	0.72	91.12
100	1,200	35	11.78	95.29	6.34	97.46
120	1,440	1	0.34	95.62	0.18	97.64
160	1,920	1	0.34	95.96	0.18	97.83
200	2,400	1	0.34	99.33	0.18	99.46
300	3,600	9	3.03	98.99	1.63	99.64
500	6,000	2	0.67	100	0.36	100

According to the investigation, there were 46.2% zero payment wishes in the sample. For this reason, the calculation model of the WTP was corrected. The formula is described as follows:

$${E'_{{\rm{WTP}}}} = E_{{\rm{WTP}}}^ + \times \left( {1 - {\rm{\mu }}_{\rm{0}}^{{\rm{WTP}}}} \right)$$ (2)

where, ${E'_{{\rm{WTP}}}}$ represents the expected value of nonnegative WTP; $E_{{\rm{WTP}}}^ + $ represents the expected value of positive WTP; and ${\rm{\mu }}_{\rm{0}}^{{\rm{WTP}}}$ represents the ratio of zero payment intention.

It can be calculated that: ${E'_{{\rm{WTP}}}}$ is 24.79 yuan/(month·household), that is, 297.48 yuan/(year·household).

If one household unit operates with three individuals, the ${E'_{{\rm{WTP}}}}$ is 8.29 yuan/(month·capita); if one household unit operates with four individuals, the ${E'_{{\rm{WTP}}}}$ is 6.20 yuan/(month·capita).

The mathematical estimation model was used to obtain the per capita ecological compensation standard for local residents (Yu & Cai, 2015). *Q* denotes the amount of ecological compensation payment, *W*_WTP_ was used to describe the maximum willingness of payment and *N* to describe the number of people who use running water in the city. Among them, 831,060 people are living in the downtown area, while 126,170 people who use the same water supplies from the Wuxijiang River are living in urban areas. *M* was used to stand for the population in the four towns of Wuxijiang River water source: Lingyang Town (5,467), Jucun Town (4,063), Huangtankou Town (9,652) and Hunan Town (11,868). All population data came from public statistics released in December of 2015. Then, the upper limit of the ecological compensation standard was given by:

$$\eqalign{& Q = {W_{{\rm{WTP}}}} \times N/M = 8.29 \times 12 \times {{831,060 + 126,170} \over {5,457 + 4,063 + 9,652 + 11,868}}  \cr  & \quad  = 2993.81\,{\rm{yuan}}/\left( {{\rm{year}} \cdot {\rm{capita}}} \right) \cr} $$ (3)

The lower limit of ecological compensation standard is given by:

$$\eqalign{& Q = {W_{{\rm{WTP}}}} \times N/M = 6.20 \times 12 \times {{831,060 + 12,6170} \over {5,457 + 4,063 + 9,652 + 11,868}}  \cr & \quad  = 2294.39\,{\rm{yuan}}/\left( {{\rm{year}} \cdot {\rm{capita}}} \right) \cr} $$ (4)

### Nonparametric estimation of the average WTA

Table 5 shows the WTA of water source ecological compensation of Wuxijiang River. The survey results of the WTA are analyzed. The expectation of the average WTA was calculated on the basis of the value of the affordable WTA, and the frequency of the WTA was given by:

$$E_{{\rm{WTA}}}^ + = \mathop \sum \limits_i^m {B_i} \cdot P_i^ + = 6591.95\;{\rm{yuan}}/\left( {{\rm{year}}\cdot{\rm{household}}} \right)$$ (5)

where *B_i_* was the amount of tender, *P_i_* was the probability of the amount chosen by the interviewee, and *m* is set to 48 in this paper.

**Table 5:** WTA of water source ecological compensation of Wuxijiang River.

WTA Yuan/(household·year)	Frequency (household)	Positive WTA rate (%)	Total positive WTA rate (%)	WTA rate (%)	Total WTA rate (%)
0	179	–	–	47.11	47.11
300	1	0.50	0.50	0.26	47.37
400	3	1.49	1.99	0.79	48.16
450	3	1.49	3.48	0.79	48.95
500	4	1.99	5.47	1.05	50.00
550	3	1.49	6.96	0.79	50.79
600	1	0.50	7.46	0.26	51.05
700	1	0.50	7.96	0.26	51.31
750	1	0.50	8.46	0.26	51.57
800	4	1.99	10.45	1.05	52.62
1,000	9	4.48	14.93	2.37	54.99
1,200	7	3.48	18.41	1.85	56.84
1,440	1	0.50	18.91	0.26	57.10
1,500	4	1.99	20.90	1.05	58.15
1,800	4	1.99	22.89	1.05	59.20
2,000	11	5.47	28.36	2.90	62.10
2,400	5	2.49	30.85	1.32	63.42
2,500	3	1.49	32.34	0.79	64.21
3,000	3	1.49	33.83	0.79	65.00
3,350	1	0.50	34.33	0.26	65.26
3,600	5	2.49	36.82	1.32	66.58
4,000	5	2.49	39.31	1.32	67.90
4,200	1	0.50	39.81	0.26	68.16
4,500	1	0.50	40.31	0.26	68.42
4,800	8	3.98	44.29	2.11	70.53
5,000	5	2.49	46.78	1.32	71.85
5,500	1	0.50	47.28	0.26	72.11
6,000	10	4.98	52.26	2.63	74.74
7,000	1	0.50	52.76	0.26	75.00
7,200	4	1.99	54.75	1.05	76.05
8,400	1	0.50	55.25	0.26	76.31
9,600	1	0.50	55.75	0.26	76.57
10,000	6	2.99	58.74	1.58	78.15
10,800	2	1.00	59.74	0.53	78.68
12,000	11	5.47	65.21	2.90	81.58
14,400	6	2.99	68.20	1.58	83.16
15,000	1	0.50	68.70	0.26	83.42
16,200	1	0.50	69.20	0.26	83.68
18,000	5	2.49	71.69	1.32	85.00
19,200	2	1.00	72.69	0.53	85.53
20,000	5	2.49	75.18	1.32	86.85
21,600	7	3.48	78.66	1.84	88.69
24,000	8	3.98	82.64	2.11	90.80
25,000	1	0.50	83.14	0.26	91.06
28,000	1	0.50	83.64	0.26	91.32
28,800	4	1.99	85.63	1.05	92.37
30,000	8	3.98	89.61	2.11	94.48
36,000	9	4.48	94.09	2.37	96.85
36,500	1	0.50	94.59	0.26	97.11
38,400	3	1.49	96.08	0.79	97.90
43,200	4	1.99	98.07	1.05	98.95
48,000	2	1.00	99.07	0.53	99.48
50,400	1	0.50	99.57	0.26	99.74
72,000	1	0.50	100.00	0.26	100
Tot up	380	100.00	–	100.00	–

Through the survey, it was found that there 47.1% of the surveyed population have a zero WTA. Therefore, according to the Spike model (Du et al., 2013) of econometrics, the computation model of WTA is corrected by:

$${E'_{{\rm{WTP}}}} = E_{{\rm{WTA}}}^ + \times \left({1-{\rm{\mu }}_0^{{\rm{WTA}}}} \right)$$ (6)

where ${E'_{{\rm{WTP}}}}$ is the expectation of non-negative WTA, $E_{{\rm{WTA}}}^ + $ is the expectation of positive WTA, and ${\rm{\mu }}_0^{{\rm{WTA}}}$ is the rate of zero WTA.

By computation, the ${E'_{{\rm{WTA}}}}$ is 3864.48 yuan/(year·household). Most of the residents in this area are farmers, and in this area, if one household includes four persons, ${E'_{{\rm{WTA}}}}$ is 3864.48 yuan/(year·household). Then, the average WTA of the local residents in Wuxijiang River was determined.

### Parametric estimation of the average WTP

STATA software (Yan, Zhang & Jiang, 2016) was used to analyze the economic factors that affect WTP and WTA in the survey (Xu, Yu & Li, 2015). Regression processing was implemented with stepwise regression analysis and the least squares method (Pan, 2014; Liu & Wang, 2017) to obtain variables with the greatest impact on WTP and WTA. Table 6 shows WTP of the interviewee and the regression results of the related variables. Table 7 shows the WTA of the interviewee and the regression results of the related variables.

**Table 6:** WTP of the interviewee in the Wuxijiang River water source and the regression results of the related variables.

Variable	(1) Forward selection stepwise regression Ln WTP	(2) Backward selection stepwise regression Ln WTP	(3) Least square method Ln WTP
Annual income	0.112*** [ 4.91]	0.112*** [4.91]	0.102*** [3.68]
Concern for the environment	−0.128* [−1.95]	−0.128* [−1.95]	−0.111 [−1.61]
Age	−0.00780** [−1.97]	−0.00780** [−1.97]	−0.00733 [−1.59]
Gender	0.196* [1.77]	0.196* [1.77]	0.197* [1.80]
Education level	–	–	0.0370 [0.71]
Occupation	–	–	−0.000680 [−0.04]
Water price	–	–	0.0538 [0.32]
Water consumption	–	–	0.0189 [0.11]
_cons	3.348*** [10.98]	3.348*** [10.98]	3.112*** [7.20]
*N*	552	552	552
*Adjusted R-squared*	0.6837	0.6837	0.149365

**Notes:**

The value in [] is the value of *t*.

* *p* < 0.1,

** *p* < 0.05,

*** *p* < 0.01.

**Table 7:** WTA of the interviewee in the Wuxijiang River water source and the regression results of the related variables.

Variable	(1) Forward selection stepwise regression Ln WTA	(2) Backward selection stepwise regression Ln WTA	(3) Least square method Ln WTA
Age	0.000130*** [2.70]	0.000130*** [2.70]	0.000115** [2.34]
Gender	0.0000445* [1.91]	0.0000445* [1.91]	0.0000487** [2.14]
Marital status	−0.0000412* [−1.77]	−0.0000412* [−1.77]	−0.0000452** [−1.98]
Family population	0.000593 [1.46]	0.000593 [1.46]	0.000607 [1.46]
Born locally	−0.0000736* [−1.82]	−0.0000736* [−1.82]	−0.000101** [−2.23]
In the main village	−0.0419** [−2.30]	−0.0419** [−2.30]	−0.0368* [−1.90]
Immigrated in five years	−	−	0.0000104 [1.23]
Education level	−	−	0.0000765 [1.13]
Number of acres of land	−	−	0.000000775 [0.52]
_cons	4.391*** [15.05]	4.391*** [15.05]	4.585*** [6.01]
*N*	380	380	380
*Adjusted R-squared*	0.896	0.896	0.88784

**Notes:**

The value in [] is the value of *t*.

* *p* < 0.1,

** *p* < 0.05,

*** *p* < 0.01.

By analyzing the regression results, it was found that annual income, age, gender and the amount of environmental concern were the main factors affecting WTP for ecological compensation. There was a positive correlation between the annual income of residents and WTP under consideration for only one influencing factor. The regression coefficient of the annual income was greater than zero, which represented the higher annual income and the greater amount of WTP. The age of the resident was inversely proportional to WTP. The regression coefficient of the annual income was less than zero, which represents older interviewees and a correspondingly smaller WTP. The greater the regression coefficient of the gender, the greater the amount of WTP. WTP of women was assigned to zero, while men were assigned to one. Thus, men had greater WTP. The “concern degree” of the environment was directly proportional to the descriptive statistics. The regression coefficient of the concern degree for the environment was less than zero, which indicated that the greater the value of the descriptive statistics, the greater the WTP.

According to the obtained regression results, the average WTP was obtained by:

$$\{ \matrix{{{\rm{ln}}{W_{{\rm{WTP}}}} = {\rm{\varepsilon }}x + {\rm{\delta }}}  \cr {{E_{{\rm{WTP}}}} = {\rm{exp}}\left( {{\rm{\varepsilon }}x + {\rm{\sigma }}/2} \right)}  \cr } $$ (7)

For the 46.2% of the population in which there was zero payment intention, the above formula needs to be adjusted as:

$$\{ \matrix{{{\rm{ln}}{W_{{\rm{WTP}} + 1}} = {\rm{\varepsilon }}x + {\rm{\delta }}}  \cr   {{E_{{\rm{WTP}} + 1}} = {\rm{exp}}\left( {{\rm{\varepsilon }}x + {\rm{\sigma }}/2} \right)}  \cr  } $$ (8)

The mathematical model was used to obtain the per capita ecological compensation standard for local residents. In this model, *x* was used to describe the main factors affecting the willingness of the respondents to pay, annual income, age, sex and ecological environmental concern for the respondents, *ε* was used to describe the regression coefficient for each factor, and *δ* was used to describe random perturbation term with a normal distribution of [0, δ/2]. According to the above, ln*W*_WTP+1_ was also a function that follows the normal distribution (Brown et al., 2014), σ represents the standard deviation of the normal distribution function. From the result of the regression, σ = 0.955205, thus;

$$\eqalign{  & {E_{{\rm{WTP}}}} = \exp \left( {C + {{\rm{\varepsilon }}_1}\overline {{x_1}}  + {{\rm{\varepsilon }}_2}\overline {{x_2}}  + {{\rm{\varepsilon }}_3}\overline {{x_3}}  + {{\rm{\varepsilon }}_4}\overline {{x_4}}  + {\rm{\sigma }}/2} \right)  \cr & \quad \quad \, = 528.72\;{\rm{yuan}}/\left( {{\rm{year}} \cdot {\rm{household}}} \right) \cr} $$ (9)

For example, in a family of three, *E*_WTP_ is 176.24 yuan/(year·capita), while in a family of four, *E*_WTP_ is 132.18 yuan/(year·capita).

The upper limit of the ecological compensation standard was given by:

$$\eqalign{& Q = {W_{{\rm{WTP}}}} \times N/M = 176.24 \times {{831,060 + 126,170} \over {5,457 + 4,063 + 9,652 + 11,868}}  \cr  & \quad  = 5434.99\;{\rm{yuan}}/\left( {{\rm{year}} \cdot {\rm{capita}}} \right) \cr} $$ (10)

The lower limit of the ecological compensation standard was given by:

$$\eqalign{& Q = {W_{{\rm{WTP}}}} \times N/M = 132.18 \times {{831,060 + 126,170} \over {5,457 + 4,063 + 9,652 + 11,868}}  \cr & \quad  = 4076.25{\rm{yuan}}/\left( {{\rm{year}} \cdot {\rm{capita}}} \right) \cr} $$ (11)

### Parametric estimation of the average WTA

Based on the analysis of relevant variables, regression analysis and the least squares method were used to regress the number of WTA with the factors related to the WTA. Based on the results, it can be seen that five variables (age, gender, marital status, whether a respondent was born locally and lived in the village) had the most influence on the respondent’s WTA compensation. Among the above factors, the regression coefficient of the age of the interviewees was greater than zero, indicating that the older the subject, the greater the value of the willingness to be paid. Putting aside the other factors, the older the respondents were, the higher their WTP for the protection of the environment. The respondents’ age had a greater impact on their WTA. Whether the family lived in the main village had a significant impact on their willingness to receive compensation. The regression coefficient was below zero, indicating that the higher the statistical value, the lower the value of the willingness to be paid. The gender regression coefficient of the interviewees was higher than zero. The descriptive statistics for female and male are zero and one, respectively, indicating that men have a stronger WTP than women. The regression coefficients of marital status and whether the respondent was born locally were less than zero, which was positively related to their willingness to receive compensation and had a negative correlation, and had less influence on their willingness to be paid. Whether the married pair was affected by the willingness to receive compensation was very small, and therefore was not tested by a level of significance test.

With analysis of the related variables, regression processing was implemented with stepwise regression analysis and the least square method for WTA values and the related factors. Regression results were used to calculate WTA:

$$\left\{ {\matrix{ {{\rm{ln}}{W_{{\rm{WTA}}}} = {\rm{\varepsilon '}} w + {\rm{\delta '}}} \cr {{E_{{\rm{WTA}}}} = {\rm{exp}}\left( {{\rm{\varepsilon '}} w + {\rm{\sigma '}} /2} \right)} \cr } } \right.$$ (12)

As there was 47.1% of the surveyed population with zero WTA, the above equation was corrected to:

$$\left\{ {\matrix{ {{\rm{ln}}{W_{{\rm{WTA+1}}}} = {\rm{\varepsilon '}} w + {\rm{\delta '}}} \cr {{E_{{\rm{WTA+1}}}} = {\rm{exp}}\left( {{\rm{\varepsilon '}} w + {\rm{\sigma '}} /2} \right)} \cr } } \right.$$ (13)

where *w* are the main factors affecting WTA, which are age, gender, marital status, if they were born locally and live in the main village, ε′ is the regression coefficient, and δ′ is a random perturbation with normal distribution of [0,σ′/2] (Rao, Lin & Kong, 2014; Li & Li, 2017). ln*W*_WTA+1_ was also a function with the normal distribution. σ^′^ is the standard deviation, and σ^′^=4.454884. Then:

$$\eqalign{ & {E_{{\rm{WTA}}}} = \exp \left( {C + {{\varepsilon '}_1}\overline {{w_1}}  + {{\varepsilon '}_2}\overline {{w_2}}  + {{\varepsilon '}_3}\overline {{w_3}}  + {{\varepsilon '}_4}\overline {{w_4}}  + \sigma \prime /2} \right)  \cr   & \quad \quad \; = 1514.04\,{\rm{yuan}}/\left( {{\rm{year}} \cdot {\rm{household}}} \right) \cr} $$ (14)

If one household includes four persons, *E*_WTA_ is 378.51 yuan/(yuan/capita·year), which was the annual per capita compensation WTA of local residents in Wuxijiang River.

## Discussion

It is necessary to try to improve the accuracy of the data, due to the lack of available data relevant to the social and economic development characteristics of the Wuxi River Basin and the ecological compensation of water sources. While obtaining primary data from on-the-spot research, several problems crop up, such as; respondents’ refusal to cooperate, respondent’s poor perception of water ecological compensation, and the failure to conduct the study in part of the basin area, thus undermining the scientific nature and authoritativeness of the survey data. In a follow-up study, organization of the investigation needs to be strengthened and more supplementary investigations need to be carried out. However, according to the results conducted by (Zheng & Zhang, 2006; Xu, Liu & Chang, 2013) based on the research data from 2015, the WTP value calculated in this paper was 74.4–176.24 yuan/(year·capita). The WTA value was 378.51–966.12 yuan/(year·capita). Income level was the most important factor affecting WTP and WTA. In 2015, the per capita disposable income of Zhejiang residents was 35,537 yuan, compared with 18,265 yuan in 2006. The ratio of the two is 1.95. Considering the increase of the per capita disposable income of Zhejiang residents, the conclusion of this paper is in good agreement with the two articles that were conducted using robust methods. Comparatively, the data of this paper is also reliable and robust.

The determination of ecological compensation standards for water sources calls for further comparison and selection. Through field investigation, we used the CVM to estimate ecological compensation standards for the Wuxi River Basin. In addition to the CVM, the ecosystem service value assessment method and the protection of the cost method were used to determine the compensation standard, without using other ways to estimate the compensation standard at the same time. More importantly, these estimation methods have many defects. It is hard to tell if finding the standard for ecological compensation estimate using the condition value assessment method is the most scientific. The effect of this ecological criterion to guide the practical compensation remains unknown. In a follow-up study, the optimization and improvement of the method for estimating the compensation standard also needs to be strengthened.

At present, it’s relatively difficult to implement the market payment method for environmental services in the water source areas in China. Wuxijiang River, a tributary of Qiantang River in Zhejiang, is an important ecological barrier upstream of Qiantang River. The Zhejiang government holds the majority of the responsibility to protect this important ecological barrier. The Wuxjiang River Dam and the Wuxijiang River Diversion Project are two strategic projects led by the government that consider both local economic and social developments, which involves a large population of residents, making the payment issue much more complex. Ecological compensation in China is typically complex, with several hierarchies of stakeholders. Since the government is playing the major role with government compensation as the major compensation method, the market compensation method accounts for a low percent. As government compensation features both limited capital support, low efficiency, and unsuitability, this leads to low and unstable benefit payments to the ecological protectors, which has become the major cause of the property in the water source area.

## Conclusion

We used the CVM to estimate the amount of compensation for subjects of ecological water supply impacts for the Wuxijiang River watershed. After in-depth analyses of the socio-economic characteristics of both subjects and the receptors, and calculation of WTP and WTA for each group, we found that the freshwater ecological compensation for the Wuxijiang River area was with the local governments at all levels and the residents of the water source area. The main loss for the residents in the water source area lies in the large amount of land that should be occupied for the development of water resources, and the limited industrial development that has resulted from environmental protection of the region. The main loss for the governments in Quzhou and the reservoir areas are the high external costs.

Despite the loss for both the residents and the local governments, these findings also suggest that there is a disagreement between the local and central government which is mainly reflected in the mismatch between the two parties in the water resources-related financial rights and powers. The disagreement among local governments is mainly reflected in the mismatch of financial power and administrative power between the upper and lower levels, produced by the administrative class. The disagreement between the people and the government in the water source area is mainly due to misunderstandings related to methods of compensation for the residents.

The resulting estimates of water source ecological compensation are as follows; using nonparametric estimation that ignored other factors, the WTP of residents in the Wuxijiang River water source was 297.48 yuan/(household·year), and the WTA was 3864.48 yuan/(household·year). The theoretical ecological compensation standard was 2294.39–2993.81 yuan/(yuan/capita·year). With parametric estimation of the other factors, WTP was 528.72 yuan/(household·year) and WTA was 1514.04 yuan/(household·year). The theoretical ecological compensation standard is 4076.25–5434.99 yuan/(yuan/capita·year). Regression analysis of socioeconomic variables of the compensation willingness and interviewees showed that annual income, age, gender, and environmental concern are important factors determining WTP. Age, gender, marital status, being locally born, and living in the main village are important factors determining WTA.

## Supplemental Information

10.7717/peerj.4475/supp-1 Supplemental Information 1 Data gathered from survey for willingness to pay for ecological compensation of Wuxijiang river basin.

10.7717/peerj.4475/supp-2 Supplemental Information 2 Raw WTA data.

10.7717/peerj.4475/supp-3 Supplemental Information 3 Questionnaire on economic and social characteristics of Wuxijiang Basin and the local residents’ response to ecological compensation.

10.7717/peerj.4475/supp-4 Supplemental Information 4 Survey for willingness to pay for ecological compensation of Wuxi River Basin.

## Additional Information and Declarations

### Data Availability

The following information was supplied regarding data availability: The raw data has been supplied as Supplemental Dataset Files.

## References

[ref-1] Arrow K, Bolin B, Costanza R, Dasgupta P, Folke C, Holling CS, Jansson BO, Levin S, Maler KG, Perrings C, Pimentel D (1995). Economic growth, carrying capacity, and the environment. Science.

[ref-2] Brown MA, Clarkson BD, Barton BJ, Joshi C (2014). Implementing ecological compensation in New Zealand: stakeholder perspectives and a way forward. Journal of the Royal Society of New Zealand.

[ref-3] Chen K (2014). Analysis of interest game in the development of western mineral resources from the perspective of stakeholders. Co-operative Economy & Science.

[ref-4] Ciriacy-Wantrup SV (1947). Capital returns from soil conservation practices. Journal of Farms Economics.

[ref-5] Costanza R, d’Arge R, de Groot R, Farber S, Grasso M, Hannon B, Limburg K, Naeem S, O’Neill RV, Paruelo J, Raskin RG, Sutton P, van den Belt M (1997). The value of the world’s environmental services and natural capital. Nature.

[ref-6] Daily GC, Söderqvist T, Aniyar S, Arrow K, Dasgupta P, Ehrlich PR, Folke C, Jansson A, Jansson BO, Kautsky N, Levin S, Lubchenco J, Mäler KG, Simpson D, Starrett D, Tilman D, Walker B (2000). Ecology: the value of nature and the nature of value. Science.

[ref-7] de Groot RS, Wilson MA, Boumans RMJ (2002). A typology for the classification, description and valuation of ecosystem functions, goods and services. Ecological Economics.

[ref-8] Du LY, Cai ZJ, Yang JM, Jiang Z (2013). Using spike model to evaluate the effect of zero response on welfare measurement—evidence from the willingness to pay for the ecological compensation of the Yangtze River Basin in Nanjing section. Journal of Natural Resources.

[ref-9] Ferraro PJ (2008). Asymmetric information and contract design for payments for environmental services. Ecological Economics.

[ref-10] Fen Y, Wang F, Yang M (2009). Research of ecological compensation Standards. Journal of Geography and Geographic Information Science.

[ref-11] Gao P, Wen YL (2004). Characters cause of poverty and its countermeasures in peripheral communities of China’s natural reserves. Research of Agricultural Modernization.

[ref-12] Gen LH, Li YY, Huang CS, Chen XY, Du X (2010). Ecological Compensation in Water Conversation and Protected Areas.

[ref-13] Guan DJ, Gong QL, Liu HM, Zheng Q (2016). Differential model establishment and its application of ecological compensation standard in the Three Gorges Reservoir of Chongqing. Acta Scientiae Circumstantiae.

[ref-14] He LP (2012). Study on the mechanism of ecological compensation in drinking water source area—taking Songhua Dam water source area of Kunming as an example. Ecological Economy.

[ref-15] Jin LS, Li XY, Zuo T (2007). Payment for environmental services: international practice and lessons and implication for China. Ecological Economy.

[ref-16] Kong F, Xiong K, Zhang N (2014). Determinants of farmers’ willingness to pay and its level for ecological compensation of Poyang Lake Wetland, China: a household-level survey. Sustainability.

[ref-17] Lai M, Wu S, Yin Y (2015). Accounting for eco-compensation in the three-river headwaters region based on ecosystem service value. Acta Ecologica Sinica.

[ref-18] Lami F, Masetti A, Neri U (2016). Diversity of Coccinellidae in ecological compensation areas of Italy and overlap with maize pollen shedding period. Bulletin of Insectology.

[ref-19] Landell-Mills N, Porras TI (2002). Silver Bullet or Fools’ Gold? A Global Review of Markets for Forest Environmental Services and Their Impact on the Poor.

[ref-20] Li G, Li X (2017). Ecological compensation standard, payment amount and adjustment target in national key ecological function areas. Journal of Xi’an Jiaotong University (Social Sciences).

[ref-21] Li YD, Chen BF (2003). The values for ecological service function of tropical natural forest in Hainan Island, China. Forest Research.

[ref-22] Liu C, Liu W, Xu M (2014). The provincial eco-compensation standard of China based on ecological value equivalents. Resources Science.

[ref-23] Liu J, Wang Q (2017). Accounting methods research for ecological compensation standard in the three-river headwaters region based on supply cost. Research of Environmental Sciences.

[ref-24] Liu YL, Xu FR, Zhang CL (2006). Model for river basin ecological compensation. China Water Resources.

[ref-25] Lu YL, Ding SB (2009). The practice of foreign ecological compensation and its reference and enlightenment for China. World Regional Studies.

[ref-26] Ouyang ZY, Wang XK, Miao H (1999). A preliminary study on the service function of land ecosystem in China and its ecological and economic value. Acta Ecologica Sinica.

[ref-27] Pagiola S, Arcenas A, Platais G (2004). Can payments for environmental services help reduce poverty? An exploration of the issues and the evidence to date from Latin America. World Development.

[ref-28] Pan J (2014). Regional eco-compensation standard in Gansu Province. Chinese Journal of Ecology.

[ref-29] Pretty J, Ward H (2001). Social capital and the environment. World Development.

[ref-30] Qiao HQ, Cheng WS, Liu XL (2016). Agricultural ecological compensation based on contingent valuation method intend and pay level evaluation. Bulletin of Soil and Water Conservation.

[ref-68] Qiao XN, Yang YJ, Yang DG (2012). Review of payments for ecosystem services and the key issues in a watershed. Progress in Geography.

[ref-31] Rao H, Lin C, Kong H (2014). Ecological damage compensation for coastal sea area uses. Ecological Indicators.

[ref-32] Research Group on China’s Ecological Compensation Mechanism and Policy (2007). China’s Ecological Compensation Mechanism and Policy Research.

[ref-33] Ruan B, Xu F, Zhang C (2008). Watershed research and practice of ecological compensation. Journal of Hydraulic Engineering.

[ref-34] Sagona W, Chirwa P, Kanyerere T, Jenya H (2016). Assessment of payment for environmental services in Zomba mountain forest catchment area, Malawi. Journal of Environmental Science and Engineering B.

[ref-35] Shen MH, Gao DK (2009). Compensational mechanism on water resource conservation. Economic Geography.

[ref-36] Shen MH, He LQ (2002). The classification of externality and the evolvement of externality theory. Journal of Zhejiang University (Humanities and Social Sciences).

[ref-37] Shen MH, Lu J (2004). On the compensation mechanism of ecological protection. Zhejiang Academic Journal.

[ref-38] Shen MH, Yang T (2004). The three theoretical cornerstones of the ecological compensation mechanism. China Environmental News.

[ref-40] Song L, Liu SJ (2005). A Collection on China’s Macroeconomy.

[ref-41] Thapa SR (2016). Opportunities and challenges of payment for environmental services: (a case study from Phewa watershed, Kaski District Nepal).

[ref-42] Turner RK, Paavola J, Cooper P, Farber S, Jessamy V, Georgiou S (2003). Valuing nature: lessons learned and future research directions. Ecological Economics.

[ref-43] Wang L, Su Y, Cui GF (2011). Quantitative study on the ecological compensation for nature reserves based on the “Virtual Land” method. Journal of Nature Resources.

[ref-44] Wang R, Wang Q, Liu N (2007). Study on the ecological compensation of the water source in the East Route of the South-North Water Transfer Project. Environmental Protection Science.

[ref-69] Wang XJ, Zhang QZ, Liu XW, Wen WJ (2010). Concept and standard of ecological compensation, and role of government: based on types of the roles of human activities to ecological system. China Population, Resources and Environment.

[ref-45] Wackernagel M, Onisto L, Bello P, Linares AC, Falfán ISL, García JM, Guerrero AIS, Guerrero MGS (1999). National natural capital accounting with the ecological footprint concept. Ecological Economics.

[ref-46] Wei C, Shen MH (2011). An econometric-based model of basin ecological compensation and application from the perspective of pollution rights. China Population, Resources and Environment.

[ref-47] World Health Organization (2005). Ecosystems and Human Well-being Health Synthesis (Millennium Ecosystem Assessment).

[ref-48] Wunder S (2005). Payments for environmental services: some nuts and bolts.

[ref-49] Wunder S, Engel S, Pagiola S (2008). Taking stock: a comparative analysis of payments for environmental services programs in developed and developing countries. Ecological Economics.

[ref-50] Xie JC, Xi BJ, Huang JM (2014). Game analysis and ant colony algorithm for water resources protection compensation in River Basin. Journal of Natural Resources.

[ref-51] Xiao JH, Wang M, Yu QD, Liu J (2015). The evaluation models of ecological compensation standard on the large-scale hydropower engineering construction based on ecological footprint: a case of Three Gorges Project. Acta Ecologica Sinica.

[ref-52] Xie XY (2003). Strengthen the Wuxijiang resources protection to ensure a clean water in Qujiang. Environmental Pollution and Control.

[ref-53] Xu DW, Liu CY, Chang L (2013). A study on the disparity of WTP and WTA of the basin’s willingness to compensate: based on the residents’ CVM investigation in the middle Liaohe drainage basin. Journal of Natural Resources.

[ref-54] Xu LY, Yu B, Li Y (2015). Ecological compensation based on willingness to accept for conservation of drinking water sources. Frontiers of Environmental Science & Engineering.

[ref-55] Xu ZM, Cheng GD, Zhang ZQ, Su ZY, John L (2003). Applying contingent valuation in China to measure the total economic value of restoring ecosystem services in Ejina region. Ecological Economics.

[ref-56] Yan J, Zhang X, Jiang M (2016). Study on Eco-compensation standard for remediation of heavy metal polluted farmland with CVM: a case study of the Dahuanjiang River Valley, Guangxi. Journal of Ecology and Rural Environment.

[ref-57] Yang L, Wang H, Sun J (2014). The construction of the marine ecological compensation system from the perspective of marine administrative planning. Journal of Ocean University of China.

[ref-58] Yang X, Cai Y, Zhang A (2017). Estimation of farmland eco-compensation horizontal transferring payment amount in Wuhan metropolitan area—from the perspective of spillover ecological value measured by choice experiment. Resources and Environment in the Yangtze Basin.

[ref-59] Yu L, Cai Y (2015). Ecological compensation based on farmers’ willingness: A case study of Jingsan County in Hubei Province, China. Chinese Journal of Applied Ecology.

[ref-60] Zbinden S, Lee DR (2004). Paying for environmental services: an analysis of participation in Costa Rica’s PSA Program. World Development.

[ref-61] Zhang W, Ming Q, Niu J (2017). Calculation and mechanisms for ecological compensation credits in the drinking water source region of plateau cities: a case study from the Songhuaba Reservoir region of Kunming. Geographical Research.

[ref-62] Zhang YF, Zhao M (2007). Review on the validity and reliability of CVM in evaluation of ecosystem service and a case design study. Advances in Earth Science.

[ref-63] Zhang ZQ, Xu ZM, Cheng GD, Su ZY (2002). Contingent valuation of the economic benefits of restoring environmental services of Zhangye Prefecture of Heihe River Basin. Acta Ecologica Sinica.

[ref-64] Zhao G, Li C, Peng P (2016). Ecological compensation standard assessment for organic chestnut production in ecologically sensitive areas—a case study on Miyun reservoir areas. Chinese Journal of Agricultural Resources and Regional Planning.

[ref-65] Zheng HX, Zhang LB (2006). Research on the standardization of compensation for the service of ecosystem in river valley. Environmental Protection.

[ref-67] Zhou Z, Sun S, Cui P (2017). Amendment of ecological compensation standards for drinking water source protection areas. South-to-North Water Transfers and Water Science & Technology.

