# Supplementary material for: Games between stakeholders and the payment for ecological services: evidence from the Wuxijiang River reservoir area in China
Source: PeerJ. 2018 Mar 8;6:e4475. doi: 10.7717/peerj.4475 (PMC5845578; doi:10.7717/peerj.4475)
Supplement: Supplemental Information 3 [file peerj-06-4475-s003.docx]

**Questionnaire on Economic and Social Characteristics of Wuxijiang Basin and the Local Residents’ Response to Ecological Compensation (Fill-In Version)**

( ) Town ( ) Village No.( )

*Please record the general information of the surveyed villages before conducting the survey: name of the villages______, altitude (meters) _____, total households _____, number of surveyed households ______, the shortest distance to Wuxijiang River(meters) _____, the shortest distance to village or town government(meters) _____.*

***Notice:*** *A. Ecological compensation refers to the economic compensation for institutions or individuals that have given up their own interests in protecting and restoring the ecological environment. B. The survey results are for research only. Thank you for sparing time to participate in this survey.*

**Side A**

| **Serial Number** |  | **Name** |  | **Contact** |  |
| --- | --- | --- | --- | --- | --- |
| **Items** | | **Description** | | | **Results** |
| Respondent’s Gender | | Male=1；Female=0 | | |  |
| Respondent’s Age | |  | | |  |
| Respondent’s Marital Status | | Unmarried=0；Married=1；Single/Widowered or Widowed=2 | | |  |
| Householder’s Age | |  | | |  |
| Householder’s Gender | |  | | |  |
| Householder’s Education Degree（schooling for how many years） | |  | | |  |
| Whether the Householders born in the current village | | （0/1） | | |  |
| Whether immigrated to the current village in the past 5 years | | （0/1） | | |  |
| Family population | |  | | |  |
| Family members with education above primary school | |  | | |  |
| Whether located in the major villages | | （0/1） | | |  |
| Land owned by the family | | Measured by “mu” | | |  |
| Crops and farmland ratio | | Percentage of farmland used for planting crops | | |  |
| Type of house | | 0=mud-wall house；1=one-story brick house；2=storied house | | |  |
| Family with modern toilets | | （0/1） | | |  |
| Family available labor force | | Including full-time and part-time | | |  |
| Family member with regular work | | At least one member=1；none= 0 | | |  |
| Total annual cash income | | （RMB） | | |  |
| Family income from agriculture | | （RMB） | | |  |
| Family income from regular work or business | | （RMB） | | |  |
| Total annual non-farm cash income | | Farm cash income=0；non-farm cash income= 1 | | |  |
| Family annual total expenses | | Family total annual expenses in the past year（RMB） | | |  |
| Total agricultural expenses | | （RMB） | | |  |
| Total labor employment expenses | | （RMB） | | |  |
| Total education expenses | | （RMB） | | |  |
| Distance to the Wuxijiang River(meters) | | estimated | | |  |
| Number of large-sized livestock | |  | | |  |
| Number of poultry | |  | | |  |
| Family total property value | | （RMB） | | |  |
| Whether the householder accepted survey or not | | （0/1） | | |  |

**Side B**

( ) Village ( ) Town No.( )

| Family Population | Family Annual Income and Constitutes | | The name and amount of annual subsidies from the government | | The name and amount of annual subsidies wished to be obtained from the government for the sake of protecting the water source | | The problems the family wishes government to help solve for the sake of protecting the water source |
| --- | --- | --- | --- | --- | --- | --- | --- |
|  |  | Yuan |  | Yuan |  | Yuan |  |
|  |  | Yuan |  | Yuan |  | Yuan |  |
|  |  | Yuan |  | Yuan |  | Yuan |  |
|  |  | Yuan |  | Yuan |  | Yuan |  |
|  |  | Yuan |  | Yuan |  | Yuan |  |
|  |  | Yuan |  | Yuan |  | Yuan |  |
|  |  | Yuan |  | Yuan |  | Yuan |  |
|  |  | Yuan |  | Yuan |  | Yuan |  |
|  |  | Yuan |  | Yuan |  | Yuan |  |
|  |  | Yuan |  | Yuan |  | Yuan |  |
|  |  | Yuan |  | Yuan |  | Yuan |  |
|  | **Family Total Annual Income** | Yuan | **Total Annual Subsidy** | Yuan | **Total Annual Ecological Compensation** | Yuan |  |

**(Thanks for the respondents’ cooperation and help.)**

**(Survey Location ______ Survey Time _____ Investigator’s Name____ Major and Grade_____ Contact Number_____)**
