# Supplementary material for: Games between stakeholders and the payment for ecological services: evidence from the Wuxijiang River reservoir area in China
Source: PeerJ. 2018 Mar 8;6:e4475. doi: 10.7717/peerj.4475 (PMC5845578; doi:10.7717/peerj.4475)
Supplement: Supplemental Information 4 [file peerj-06-4475-s004.docx]

**Supplemental Files 4: Survey for Willingness to Pay for Ecological Compensation of Wuxi River Basin**

*(Please make clear to the public about the following three points)*

*A. Water is the foundation of life. Drinking water safety is the basic requirement of the people, and the safety of drinking water is the premise of sustainable ecological environment protection. We drink Wuxi River water, and in order to protect its water, people in the mountainous area along the river have paid a lot.*

*B. Ecological compensation refers to the economic compensation for units and individuals who making sacrifices for protecting and restoring the ecological environment and its functions.*

*C. The results of this questionnaire are only used for scientific research. Thank you very much for your participation in this questionnaire survey!*

The following is the formal contents of the survey, which is given to the public to select and find the answers. Method: fill in above the horizontal line; add the tick (√) for the single option; fill in the bracket ( ).

1. Your gender (male, female), age ( ), and education ①primary school；②junior middle school ③high school (including technical secondary school and vocational school); ④associate; ⑤bachelor; ⑥ master and above.

2. Your occupation: ①state organs, party organizations, and directors of enterprises and institutions; ②technical personnel; ③the clerk and the relevant personnel; ④commercial staff; ⑤service staff; ⑥forestry, animal husbandry and fishery workers ⑦ civil servants, teachers and journalists; ⑧medical personnel, lawyers and finance practitioners; ⑨ soldier; ⑩other

3. Your family income is about ：①20,000 Yuan per year; ②20,000 to 30,000 Yuan; ③30,000 to 40,000 Yuan; ④40,000 to 50,000 Yuan; ⑤50,000 to 60,000 Yuan; ⑥60,000 to 70,000 Yuan; ⑦70,000 to 80,000 Yuan; ⑧over 80,000 Yuan

4.What is the price of water for residents? □I don't know; □I know, it's about _____Yuan/ton.

5.What is the water consumption of your family for a month? □I don't know; □I know, it's about _____ ton.

6. To what extent you are concerned about the environmental damage problem. ①very concerned ; ②be more concerned about important environmental event; ③pay attention occasionally; ④pay little attention

7. In your opinion, the percentage of global diseases related to water is: ①20%; ②50%; ③ 60%; ④ 80%

8. In your opinion, the percentage of the global child deaths caused by drinking water problems is: ①10%; ② 30%;③ 40%; ④ 50%

9. Do you understand the concept of "ecological crisis", "safe drinking water" and "ecological compensation"? ①I know very well what is going on; ②I have heard of such concepts, but I do not quite understand what is going on; ③I have not heard of it

10. Do you think the Wuxi River Basin now needs to deal with ecological destruction and environmental pollution? ①very urgent; ②pressing; ③acceptable; ④there is no need for improvement

11. Do you think the ecological environment changes in the upstream of Wuxi River, whether it is getting worse or better, and the relationship with you is big? ① it has nothing to do with me; ②it affects my life, but not too much; ③a bad environment can affect my life, but it won't do me any good if it gets better. ④the change in ecological environment is very important to my own life

12. Do you think which is more effective to solve the ecological environmental protection problem in the upstream of Wuxi River? ①an executive order; ②laws and regulations; ③economic compensation; ④ other

13. If economic compensation is to be made to the upper reaches of Wuxi River, who should compensate for it: ①the central government ②the local government of downstream Wuxi River; ③the user of Wuxi River; ④all ecological beneficiaries

14. If upstream region of Wuxi River, in order to achieve the improvement of the whole river basin's ecological environment, strengthens the ecological construction and ecological recovery, to encourage the upstream region of these contributions, will you take out some money from your personal income to support? ①not willing; ②willing

**☆**If you choose not willing, your reason is: ①family income is low;② not interested in the ecological environment;

③the upstream regional effort will not achieve the desired objectives; ④environmental changes have little impact on individuals; ⑤It is the duty of the government to be funded by the state and should not be paid by individuals and families.⑥other (please add)______________________

**☆**If you choose "willing", then answer questions 15-16:

15. What is the maximum amount of money your family is willing to pay per month? (add the tick "√ "in the box )

□ 0 Yuan □ 20 Yuan □ 40 Yuan □ 60 Yuan □80 Yuan □ 100 Yuan □ 120 Yuan □ 140 Yuan □ 160 Yuan □ 180 Yuan □ 200 Yuan □ 220 Yuan □ 240 Yuan □ 260 Yuan □ 280 Yuan □ 300 Yuan □ 320 Yuan □ 340 Yuan □ 360 Yuan □ 380元Yuan □ 400元Yuan □ 420 Yuan □ 440 Yuan □ 460 Yuan □ 480 Yuan □ 500 Yuan

16. If it is a one-time fundraising, you will take out________ Yuan (RMB) from your family annual income to fund it.

17. How would you like to pay? Please choose: ①donate money;②pay the ecological environmental protection tax; ③pay water bills; ④work for money

18. If you are a conservationist of upstream Wuxi River (your career or family production and business operation activities are good to Wuxi River basin ecological environment's recovery and protection), do you want to get the compensation amount is: ①to meet individual basic life need; ②to reach the average level of the upstream region; ③the average level of the middle and lower reaches; ④the equivalent of giving

19. If you are an ecological protection organization upstream of Wuxi River, you hope that the compensation for ecological protection will be: ①to maintain normal ecological protection costs; ②A slight increase in the maintenance of normal costs; ③a further increase in the maintenance of normal costs;

Thank you very much for your cooperation and help.

(Place for survey_________ time_________ investigator__________

major__________ grade___________ contact number___________
